# Supplementary material for: Are carbon emissions trading and green financial instruments synergistic? -Comprehensive quantitative research based on content analysis
Source: PLoS One. 2024 Mar 7;19(3):e0298601. doi: 10.1371/journal.pone.0298601 (PMC10919601; doi:10.1371/journal.pone.0298601)
Supplement: S1 Data — (ZIP) [file pone.0298601.s001.zip › Data/地级市Z_F工作报告.zip!/╝¬┴╓/┴╔╘┤╩╨/└┤╫╘╣1⁄2╓┌║┼í╛┬φ┐╦╩2╛▌═°í┐_2019.docx]

各位代表：

　　现在，我代表市政府向大会报告工作，请予审议，并请市政协各位委员提出意见。

　　一、2018年工作回顾

　　过去的一年，是辽源转型发展中矛盾倍增、挑战空前、很不平凡的一年。面对复杂严峻形势和叠加累积任务，在市委的坚强领导下，市政府与全市干部群众一道，认真学习贯彻习近平新时代中国特色社会主义思想和党的十九大精神，主动适应新时代新常态，坚持稳中求进工作总基调，积极应对新情况，妥善化解新问题，各项工作取得新进展。

　　发展基础得到夯实。妥善应对经济持续下行、指标基数调整、自然灾害等客观因素，全力破解产业、企业等实际困难，保持稳定增长的基本面。预计地区生产总值增长1%，规模以上工业增加值增长1%，地方级财政收入增长2.6%，固定资产投资增长3%，社会消费品零售总额增长4%，外贸出口额增长1.3%。物价总体水平保持稳定。农业经济健康发展。持续深化农业供给侧结构性改革，调减籽粒玉米21.4万亩，新增棚室面积9620亩，高标准农田占全市耕地30%，农作物综合机械化率达到83.1%。全力降低旱灾影响，粮食产量达到40.5亿斤。全市土地流转占比49%，居全省前列。农民专业合作社发展到3038个，农产品加工企业超过500户，17个省级农业产业化项目加快建设。新认定“三品一标”202个。东丰县被确定为“全国第三批基本实现农作物生产全过程机械化示范县”。东辽县被农业部列入全国农村一、二、三产业融合发展先导区创建名单。工业经济企稳向好。预计农产品深加工、纺织袜业、新能源产业产值分别增长10.1%、15.8%和13.4%。50户重点企业产值增长16.4%，拉动工业经济增长15.3个百分点。加强经济运行协调和要素配置保障，城投集团助企融资超过60亿元，向省争取专项资金近8000万元，政银企对接为210户企业直接贷款15亿元。经济开发区被列为年度直购电试点园区，辽矿集团增量配电试点项目启动区域划分。“双停”企业脱困和“僵尸企业”清理纳入重要日程。服务业显现活力。实施5000万元以上服务业项目20个，服务业投资、增加值分别增长30%和4%。电子商务、文化旅游、品牌运营等业态加快发展。“1688”电商服务中心辽源站投入运行，农村电商服务站覆盖率达到60%以上，东北袜业电子商务园区线上销售突破11.2亿元。鴜鹭湖被评为国家级湿地公园，凤鸣湖试点规划调整获得国家批准。全市旅游收入增长20.2%。成功举办中国辽源第四届国际袜业交易会。铝型材、袜制品出口分别增长38%和23%，“国际贸易单一窗口”业务覆盖率达到72%。

　　新旧动能加速转换。深度对接国家政策，坚持打基础、利长远，持之以恒推进高质量发展。产业布局不断优化。科学评估修编“十三五”规划及相关专项规划，谋划新能源汽车配套基地、轨道客车配套基地、梅花鹿产业振兴、冰雪装备制造等7大产业工程。启动智慧辽源建设，扎实推进“六大体系”“十大工程”，完成东北卓越云二期工程，建设“政务云”平台，新龙塑胶、鑫锐机械等18户企业列入省级两化深度融合项目库。重点项目扎实推进。开工建设5000万元以上项目127个，完成投资72.3亿元。吉林诺德高科年产1.35万吨高性能树脂、金州现代农业园区冷链物流、莎普爱思生产扩建等项目达产见效，鸿图锂电隔膜三期、辽源国际商贸物流城等新建项目扎实推进。实施工业转型升级重点项目120个。滚动储备重大项目200个以上。科技创新成果显著。建立吉商产业创新发展研究院和吉林大学辽源技术转移中心，经济开发区被工信部确定为全国产业集聚区域品牌建设试点地区和新型工业化示范基地。实施省科技项目54个，新认定省级科技小巨人企业10户，评定国家级高新技术企业、星创天地和省级科创中心7个，新建院士工作站1个。49户中小企业纳入国家科技型中小企业库。全市有效专利拥有量达到737件。辽源国家农业科技园区通过科技部认定。

　　改革开放步伐加快。聚焦破解体制机制障碍，大胆先行先试，持续释放改革红利。重点领域改革成效明显。《辽源市机构改革方案》经省委、省政府批准并开始实施。“只跑一次”改革领跑全省，一次办理比例达到92.4%，平均压缩办事时间85%以上。深化商事制度改革，新登记市场主体11458户，增长46.9%，新增小微企业1882户。“12345”市民服务热线荣获全国政府服务热线稳步攀升奖，“权力下沉+综窗服务”等5项经验在全省推广，不动产房屋交易办理全省领先，公积金贷款业务办结时限全国第一。完善防范化解金融风险体制机制，加强债务预算管理，严格规范金融市场交易，城投集团运营效率不断提高，在化解债务风险方面发挥重要作用。国有林场改革获得省级优秀，集体产权制度等农业改革扎实推进。企业年报公示率连续三年全省第一，我市综合信用指数晋位升级。供销合作社综合改革试点通过验收，东丰供销电商“双网双轨双融合”发展模式在全国推广。对外交流合作不断扩大。与绍兴缔结为友好城市，达成战略合作协议10余个，对口合作取得实质进展。积极融入“一主六双”空间发展布局和“长春经济圈”产业产品合作，建立完善与国内外重要商会、协会对接机制。锁定环渤海、长三角、珠三角等重点区域，对接中广核、双汇集团、中国核电、北汽福田、万达集团、华为集团等大型企业，新入省调度系统项目60个，项目履约率 93.33%，引资到位率45.62%。

　　生态治理扎实推进。坚决扛起政治责任，上争下促，统筹施策，重点难点遗留问题得到有效化解。突出打好东辽河流域污染防治攻坚战。全面落实习近平总书记重要批示精神，修编落实东辽河流域水环境保护与治理规划，围绕水生态修复、水污染防治、水资源保护，开工建设东辽河流域污染治理项目24个。启动市污水处理厂、东北袜业工业污水处理厂提标扩容改造，加快推动市新建污水处理厂PPP项目，开工建设平岗镇、渭津镇、金州乡生活污水处理项目。仙人河截污干管建设工程基本完工，封堵入河排污口46个。城市雨污分流改造完成23.56公里。东辽河流域禁养区内规模养殖场全部关停或搬迁，畜禽粪污处理设施实现全覆盖。建设人工湿地14万平方米，人工造林2.69万亩，治理裸露山体159处。东辽县被评为全国绿化模范县。着力实施环境突出问题专项整治。中央环保督察反馈的25个问题销号完成11个，化解信访问题945件。推进大气污染防治，实施工业脱硫脱硝治理项目14个，燃煤小锅炉淘汰率达到99.3%，黄标车淘汰率达到77.9%。秸秆禁烧实现零火点，秸秆综合利用率达到75%。全年空气质量优良天数达到317天。推进全域城乡生活垃圾一体化处理，市区垃圾无害化处理率达到95%，90%的行政村实现垃圾集中处理。成功创建节水型城市。启动土壤防污防治计划。严厉打击环境违法行为，关停“散乱污”企业11户，立案查处环境违法案件107件。

　　城乡面貌不断改善。着力破解征拆、资金等难题，加快补齐城市建设欠账。推进基础设施建设。伊开高速二期接近尾声，集双高速辽源段启动建设，完成国道饶盖线、集阿公路养护改造，修建四好农村公路500公里，完成安全生命防护工程235公里。实施东辽河城防闭合工程。推动“引松入辽”辽东支线建设。铺设“气化辽源”燃气管线31.2公里，新增管道天然气用户1.5万户。加强城市管理。城市建设管理提挡升级攻坚顺利收尾，开展城市管理“六大行动”，制定城市绿地、停车场等规划，建立智能化城市道路指挥系统。完成实验中学西段拓宽改造，实施仙城大街、东吉大路亮化工程，新建一批水洗公厕，完成宠物市场迁移。新建改造景观带6条，提升街头绿地20处。街路机械化清扫率超过60%。划定居民小区停车位2272个。保持“禁烧”“拆违”常态化，拆除违建2.1万平方米。我市顺利通过省级卫生城复审。统筹城镇化建设。确定新一轮省级重点村工作，完成金州乡省级示范城镇居民安置区工程。实施农村人居环境三年整治行动计划，扎实推进重点镇完善基础设施和公共服务，改造农村危房1938户，改厕5780户。开展“大棚房”专项整治行动，整改一类二类问题8个。

　　民生工程深入实施。坚决保障民生支出，回应群众期盼，办结惠民实事69件。脱贫攻坚取得实效。投入扶贫资金6077万元，实施扶贫项目52个。改造危房976户。建设饮水安全工程47处。义务教育阶段建档立卡贫困家庭适龄人员入学率100%。“先诊疗后付费”等健康扶贫政策受益13382人（次）。建立健康扶贫“五道防线”，贫困人口实际报销比例提高到90%。完善“五点双享两救助”兜底保障机制，新增受益人口1685人。17个贫困村出列、2123人脱贫年度目标全面完成，贫困发生率降到0.54%。群众获得感显著增多。城镇新增就业3.5万人，农村劳动力转移就业17.3万人（次），城镇登记失业率控制在3.02%。征缴企业职工基本养老保险费18亿元，达到历史最好水平。城乡低保、医疗救助、特殊家庭扶助、残疾儿童救助标准进一步提高，惠及群众5.7万人。城乡教育“四个统一”标准、“两免一补”政策全覆盖，新增普惠性幼儿园20所，现代学徒制试点顺利通过教育部验收，现代职教园区一期工程投入使用。公立医院改革代表全省通过国家考核，所有药品实现零差率销售，设备检查收费下降15.5%，家庭医生签约服务49.6万人。医养结合模式全面推广，全市62家养老机构与医疗机构全部签订服务协议。新建和货币化安置保障房550套，回迁居民5824户，解决逾期未安置居民1420户，“无籍房”不动产登记1.87万户。更新新能源公交车56台，开通延伸一批公交线路。积极创建国家级公共文化服务体系示范区，成功举办首届市民运动会，第三届辽源国际琵琶文化艺术周活动在北京国家大剧院开幕，新增一批全民健身场馆设施。平安辽源建设深入推进。强力推进扫黑除恶专项斗争，破获涉黑组织、涉恶类犯罪团伙13个，刑事案件、命案、“两抢”案件分别下降50%、5.6%和9.1%，我市连续三年被评为全国社会治安综合治理创新优秀城市。法治信访纵深推进，信访积案化解取得积极进展。统筹开展安全生产治理年与“利剑执法”行动，全市安全事故下降36%。连续34年无重大森林火灾。食药监破获部督案件4起，东丰县被确定为首批“国家餐饮服务食品安全示范县”。积极推进国防教育和双拥共建。民族团结、宗教和顺局面不断巩固。《辽源年鉴》获得国家级特等奖。广播电视、外事侨务、档案、气象、地震、人防等工作和妇女儿童、老龄、残疾人等各项事业全面发展。

　　政府建设进一步加强。自觉接受市人大法律监督、工作监督和市政协民主监督，办理人大代表建议和政协提案190件，办复率达到100%。持续推进依法治市和法治政府建设，加强“七五”普法，严格执行宪法宣誓制度。落实全面从严治党要求，牢固树立“四个意识”，坚定“四个自信”，做到“两个坚决维护”，深入开展干部作风大整顿活动，建立和修订政府工作规则等系列工作制度。推进公共资源交易电子化，政府采购节支率达5.65%。严格落实中央八项规定精神，严查重处一批“四风”和侵害群众利益问题。

　　各位代表，过去的一年，我们在前所未有的困难和挑战中奋力攻坚、砥砺前行，顶住了极端压力，筑牢了发展根基，积蓄了强大动力，取得了来之不易的宝贵成果。这是省委、省政府和市委正确领导、科学决策的结果，是市人大及其常委会、市政协和社会各界有效监督、大力支持的结果，是全市人民奋发有为、团结拼搏的结果。在此，我代表市政府，向奋斗在各条战线上的广大干部群众，向历任老领导、离退休老同志，向各民主党派、工商联、无党派人士和人民团体，向驻辽部队、中省直单位及所有关心支持参与辽源发展建设的社会各界人士、海内外朋友致以衷心感谢和崇高敬意！

　　同时，我们也要清醒地看到差距和问题，主要是：经济结构不优，体制机制不活，内生动力不足，发展方式和质量效益还有待进一步提升；主导产业不强，特色产业不优，科技、人才、品牌等仍然存在短板，产业链和集群竞争优势尚未有效形成；面对经济下行压力、创新发展要求和要素制约瓶颈，中小企业普遍遇到困难，部分重点企业更是面临难关；财政收支矛盾突出，政府债务负担沉重，城乡建设、生态环保、民生改善、公共服务、社会治理等领域面临一系列亟待解决的突出问题；部分干部和公职人员的思想不够解放，工作作风、精神状态、创新意识、担当精神、专业能力与新形势要求还有一定差距，服务企业、服务群众还不到位、不精准。对于这些问题，我们将下定决心，采取有力措施加以解决。

　　二、2019年工作安排

　　我们正处在决胜全面小康的关键征程，即将迎来新中国70周年华诞。全力推动高质量发展，建设幸福美好辽源，是市政府落实党委要求，不负人民期待，必须深入把握的主题主线。主动求为、担当作为、竞进有为，是市政府全面深化工作，必须具备的精神状态。必须主动在国家政策导向中抓机遇，切实把中央减税降费等红利用好，把振兴东北等政策用足；必须主动在全省发展大局中找定位，顺应吉林“三个五”战略、“一主六双”空间布局，谋划、扩大辽源发展新优势；必须主动在产业升级趋势中迎风口，对面向未来的人工智能、物联网等新兴产业尽早吸纳培育，积极促进传统产业的“智能+”“智慧+”；必须主动在深挖资源禀赋中求突破，用好转型城市的工业遗产、矿业资产，重视互联网“可视化”发展态势下的特色资源；必须主动在满足群众诉求中强服务，把以人民为中心的发展思想，落实为群众的所获、所感、所见；必须主动在解决突出问题中促改革，立足于辽源“一个突出五个双重”的最大实际，着手打造更加优良的发展环境。

　　今年政府工作总的要求是：以习近平新时代中国特色社会主义思想为指导，深入贯彻党的十九大和十九届二中、三中全会精神，全面落实习近平总书记在深入推进东北振兴座谈会上的重要讲话和视察东北时的重要指示精神，认真按照中央和全省经济工作会议作出的决策部署，紧紧围绕市委七届六次全会确定的目标任务，牢牢把握稳中求进工作总基调，坚持新发展理念，坚持高质量发展，持续深化供给侧结构性改革，落实“巩固、增强、提升、畅通”要求，继续打好三大攻坚战，统筹抓好稳增长、调结构、促改革、惠民生、防风险等工作，努力形成经济向稳、产业向强、环境向优、农业向精、城市向美、民生向实、发展向好、格局向宽、队伍向上的良好态势，奋力开创辽源高质量发展新局面。

　　2019年全市经济社会发展的主要预期目标是：地区生产总值、规上工业增加值、固定资产投资均增长3%左右，财政收入、社会消费品零售总额、外贸进出口总额保持平稳增长；城乡居民收入与经济增长基本同步；居民消费价格涨幅在3%以内；城镇新增就业1.5万人以上，登记失业率控制在4.5%以内。节能降耗等约束性指标完成省计划。重点抓好九项工作：

　　（一）壮大实体经济，促进经济“向稳”。

　　以稳为先，提振实体经济，防止出现波动。以稳求好，推动转型增效，实现高质量发展。

　　抓投资稳增长。深入开展“项目建设年”活动，制定实施重点建设项目和政府投资项目计划，着力推进5000万元以上项目123个，谋划储备亿元以上项目140个以上。围绕推动产业裂变式发展，重点抓好重庆三圣医药制造基地、国际梅花鹿产业创投园等52个产业类项目；围绕提升区位优势、完善城市承载功能，启动推进高速铁路、城市道路桥梁等21个基础设施类项目；围绕补齐生态环保、健康养老、文教体育等领域短板，重点推进中医大厦、十九中学异地新建等38个民生公益类项目；围绕完善服务业设施和体系，重点实施欧亚体验式购物广场、辽源国际商贸物流城等12个服务业项目。围绕工业供给体系优化升级，谋划实施日新智能针织装备及关键零部件制造等60个重点技术改造项目。把项目建设作为“一把手”工程，完善“三大员”机制，落实清单式服务措施，推动市新建污水处理厂、诺德高科年产1万吨太阳能光伏膜等“三早”项目如期开复工，亿达碳纤维材料深加工、华纺无油剂静电功能短纤维等在建项目提早投达产，确保所有开复工项目按序时进度形成实物工程量。

　　抓工业稳增长。出台针对扩总量、盘存量、引增量的政策措施，谋划建立研发中心、设计中心、品牌中心和电商中心，促进工业提速增效。加强工业运行监控，保障生产要素供给，推动50户重点企业稳定生产。全力帮助利源精制、麦达斯铝业走出困境，保障正常生产经营，巩固提升支撑能力。完善“双停”企业盘活机制，落实领导包保和“一企一策”措施，推动企业转型转轨、兼并重组，有效盘活存量资源。开展“个转企”“小升规”专项行动，建立奖励办法和专项资金，整体提升企业数量质量体量，全年新增小微企业500户以上。实施“专精特新”中小企业培育计划，加快打造一批“单项冠军”和“小巨人”企业，带动上下游产业链协同发展。

　　抓消费稳增长。设立辽源“城市节”，持续开展“千企促销”、名优产品推介活动，丰富展会、节庆等主题消费，拓展农村消费空间，不断活跃传统消费市场。培育新的消费热点，打造辽源国际商贸物流城、中农农副产品交易市场等精品集聚区，创建纺织袜业、寒葱顶影视文化等省级特色小镇，加快园区消费模式创新。实施全域旅游发展计划，积极融入省里产业空间布局，丰富乡村体验、休闲养生、红色文化、冰雪娱乐等特色旅游产品，规划建设旅游商品研发生产基地。

　　抓出口稳增长。筹建“辽源海关”，不断扩大对外贸易规模。抓好跨境电商平台建设，巩固欧洲、韩国等传统市场，开辟“一带一路”沿途国家新兴市场，促进辽源地产品线上线下销售。依托三大出口基地，扩大东北袜业、唐豆豆等企业直接出口规模，增加铝型材、松子仁、棉袜、明胶、蛋品等产品国际市场份额，让辽源制造不断走向世界各地。

　　（二）注重创新驱动，促进产业“向强”。

　　加快结构调整步伐，创新、整合、提升产业基础优势，推动制造业高质量发展。以2019年为起步年，力争用3年左右时间，纺织袜业达到200亿级规模，蛋品+特色农产品加工、冶金建材产业达到100亿级规模，高精铝加工、梅花鹿+医药健康、新能源汽车配套产业达到50亿级规模，形成“211555”产业格局。

　　提升科技创新能力。以企业为主体，以高校院所为支撑，加快协同创新步伐。推进与中科院长春“三所”、吉林大学、长春理工等院校合作，力争在华纺静电高性能环保材料关键技术研究、鸿图锂电高品质电池隔膜研发、格致汽车工业模具智能化制造技术研发等方面实现突破。争取实施省级科技项目20个，院士工作站发展到5个，众创空间、科技示范园区增长10%，新认定国家高新技术和省、市科技“小巨人”企业10户以上。

　　提升融合发展能力。深入推进智慧辽源建设，推动互联网、大数据、人工智能与实体经济深度融合，促进数字信息技术在产业发展、政务服务、改善民生等领域广泛应用。启动科技大数据库和中小企业线上信息库建设，推广网络协同制造、智能生产、绿色制造等模式，加快云动力、元隆达等企业与机器人、数控机床等行业融合应用和模式创新。加快推进与中广核战略合作，推进秸秆等农业废弃物综合利用，打造循环经济产业典范。全面加快农产品深加工、蛋品加工、医药健康、梅花鹿、冶金建材、汽车零部件、新能源等产业的升级整合，构筑新的竞争优势。

　　提升品牌引领能力。以东北袜业园为依托，实施“一厂一品”+“100户品牌企业”创建工程，打造辽源轻纺品牌，以高端化、个性化产品抢占国内外市场。以打造北方高精铝加工基地为目标，释放我市高精尖铝制品品牌效应，推动轨道交通车体产品向汽车轻量化及更广领域延伸。以融入医药健康产业走廊平台为契机，发挥东丰梅花鹿入选我省十大特色产品优势区作用，扩大“马记鹿茸”等品牌影响，推动鹿产品向医药领域拓展。以增强农产品精深加工能力对接“长春经济圈”，放大厚德食品的源头控制、加工能力优势，借助与双汇集团合作渠道，打造中国“蛋品之都”，带动蛋品加工产业全链条、绿色化、精深化发展。以完善新能源产业链为切入点，引进战略投资者，打造吉林省新能源汽车配套基地，在锂电池、高性能隔膜、电机电控等领域形成一批区域性知名品牌。

　　提升园区承载能力。完善经济开发区体制机制，推动权力职能下放，强化平台服务功能，加强投资、审批、信用、监管等体系建设，整合两区工业集中区，系统设计产业功能区、服务业集聚区等区划，精准推进高精铝加工、纺织袜业、新能源汽车等产业布局，争创国家级经济开发区。统筹提升两县开发区承载功能，差异化设计产业发展重点。规划建设冰雪装备特色产业园区，全力支持东北袜业争创“国家级电子商务示范基地”。

　　（三）全面深化改革，促进环境“向优”。

　　以打好重塑环境攻坚战为引领，从解决深层矛盾和共性问题入手，积极稳妥推进各领域改革。

　　深化精细服务。统筹推动“放管服”改革、“只跑一次”改革与党政机构改革，按照“只进一扇门、只跑一次”要求优化机构设置和职能配置。实行市场准入负面清单制度，推进企业登记全程电子化和“证照分离”等商事制度改革。推行“互联网+政务服务”，建设政务信息资源共享交换平台和覆盖全市的协同办公平台，率先推动信用体系、公共资源交易、12345市民服务热线等重点领域数据共享交换服务，建立全市统一、入口一致的网上服务大厅，让企业和群众办事像网购一样方便。

　　优化营商环境。集中开展软环境攻坚，清理不适宜的规范性文件，打破“隐性壁垒”，在市场准入、审批许可、经营运行、招投标等法律规定范围内的各个领域中一视同仁、公平公正，重点开展窗口服务质量、涉企“四乱”、政府和企业失信专项整治。建立市、县领导联系服务企业机制，健全企业家参与政策制定机制，开展局长行长科长“三长”进企业活动，努力构建亲清政商关系，让良好的发展环境成为辽源吸引投资、汇聚人气的城市名片。

　　强化政策扶持。制定我市促进民营企业高质量发展的意见，建立税收优惠清单、涉企收费清单，高效落实企业税费减免政策。设立民营经济发展专项资金和民营企业产业创新发展基金，制定出口创汇贡献企业奖励政策。强化城投集团助企融资功能，筹建企业互助资金。积极弘扬企业家精神，依法保护企业家财产权、创新权、自主经营权，加强新一代企业家培育，打造支撑创新转型发展的生力军。

　　（四）推动乡村振兴，促进农业“向精”。

　　以农业强、农村美、农民富为愿景，科学编制全市乡村振兴规划，不断完善现代农业“三大体系”，加快建设农业精品市和畜牧业强市。

　　培育农业特色产业。加强生产体系建设，改造高标准农田30万亩，合理调减籽粒玉米，扩大绿色水稻、有机蔬菜和杂粮、经济作物种植面积。重点抓好中德东辽河源头现代生态农业示范区、东辽国家农业科技园区、东丰佰晟源食用菌产业园等现代农业产业园区建设。加强产业体系建设，突出“一乡一业、一村一品”，引领一二三产业融合。东丰县突出发展梅花鹿产业，依托睿康生物、鹏翔牧业等农产品加工企业，建设梅花鹿+医药健康产业基地。东辽县突出发展蛋品加工产业，挖掘黑猪、柞蚕等特色资源，建设蛋品+特色农产品加工产业基地。加强经营体系建设，支持农民通过土地经营权持股、项目合作、土地托管、入股等方式开展适度规模经营，鼓励龙头企业为农户提供技术培训、贷款担保、农业保险资助等服务，新增农业新型经营主体500个。加强营销体系建设，促进农业领域社交电商、内容电商、直播电商发展，带动农产品销售。

　　打造乡村优美环境。深入开展农村人居环境整治三年行动，继续治理农村生活垃圾，梯次推进生活污水治理，大力实施厕所改造，加快畜禽粪污处理配套设施建设。全面加快“大棚房”专项清理。加快农村饮水安全巩固提升、新一轮农村电网改造升级、“四好农村路”等基础设施建设。统筹推进水土流失治理、湿地恢复、绿化美化工程，大力实施环村林带建设，创建一批美丽乡村。

　　提升农民文明素质。加快改善农村公共服务，促进教育、卫生等公共资源向基层倾斜。健全乡村公共文化服务体系，加强基层综合文化服务中心建设。深入开展移风易俗行动，弘扬优秀传统文化，推进精神文明创建活动。大力培养农业农村人才，支持鼓励农民工返乡、能人回乡、市民下乡、企业兴乡创业创新。支持两委班子和干部队伍建设，确保有人干事、有钱干事。加强自治、法治、德治，建设平安乡镇、平安村庄。

　　推进农业农村改革。积极深化农村集体产权改革、三权分置改革、集体经营性资产股份合作制改革，落实国家农村土地征收、集体经营性建设用地入市、宅基地制度改革政策，加快推进国有林场改革后续工作，推广全域性供销综合改革。积极争取国家新型城镇化综合试点，加强特色小镇培育，落实县域经济发展促进政策，为乡村振兴不断注入活力。

　　（五）加强建设管理，促进城市“向美”。

　　坚持“精准规划、精致建设、精细管理”，有序推进中心城区改造和新城区开发，科学经营城市，提升城市品味，彰显城市魅力。

　　完善城乡规划体系。强化“规划先行”理念，落实“多规合一”要求，全面建立完善城市规划体系，启动编制《国土空间规划》，完善城市总体规划，加快编制重点区域控制性详细规划，完成各类专项规划。突出辽源“水在山中、山在城中、城在山水中”的自然形态，编制城市景观风貌专项规划。精准确定功能定位，完成南部新城和采煤沉陷区控制性详细规划编制。完善特色乡镇建设规划，编制46个乡村规划。制定和落实经营性用地做地计划，完善土地收储和出让方式，实现土地资源高效利用。

　　加快基础设施建设。启动四通高铁项目前期工作，争取辽源机场项目完成选址批复，城市大三环项目列入省规划，伊开高速二期、集双高速辽源段建成通车。加快东辽河城市防洪闭合工程，推进仙人河老工业区搬迁改造创建全国示范工程。实施福镇大路、财富北路等22条街路改造，有计划地消除“断头路”。改造各类管网67公里。新建改造一批景观带、绿地小品，栽植绿化苗木60万株。维修改造龙首山公园、向阳山森林公园以及城市广场，完善矿山湿地公园开发设计，打造鹿鸣湖区域景观。

　　实施精细化管理。深入推进城市管理效能提升三年行动，持续开展“六个专项行动”。重点整治店外经营、占道经营、乱摆乱放等违规行为，保持街路干净整洁；完善交通渠化和信号系统设施，强化组织管理，保持街路畅通；加大拆违力度，拆除仙人河两岸、中心城区和老旧小区违法建筑设施；整体推进主要街路、居民小区绿化品质提升；实施重点街区美化工程，对沿街楼宇立面牌匾进行规范整治；实施沿河亮化工程；加强城市管网建设改造，保证管网安全稳定运行。启动智慧管理与运营中心建设，推动基础设施、安全监控、应急协同等重点领域项目应用，提高城市智能化管理水平。

　　（六）增进人民福祉，促进民生“向实”。

　　自觉践行以人民为中心的发展思想，回应群众期盼，严格落实九项惠民工程，制定细化惠民实事行动计划，不断提升群众获得感、幸福感、安全感。

　　完善社会保障体系。强化大学生、退役军人、农民工、下岗职工等重点群体就业援助，落实创业培训小额贷款、创业贴息政策，城镇新增就业1.5万人，转移农村劳动力15万人（次）。加强社会保险扩面征缴，继续提高养老金、失业金和城乡低保标准，深化机关事业单位养老保险制度改革。探索建立救灾、救助、救济一体救助模式。加强社区居家养老设施配套建设，鼓励支持“医养融合”养老机构发展，加快形成以居家养老为基础、社区养老为依托、机构养老为补充的养老服务体系。实施棚户区改造收尾攻坚，完成5.06万户“无籍房”不动产登记，加快化解棚改逾期未安置问题。规范整治物业服务行为，实施10个老旧小区综合整治和“三供一业”工程改造。

　　加强优质公共服务供给。加快全国第四批公共文化服务体系示范区建设，深入开展文化进村镇、进社区活动，拓展街头巷尾文体活动空间。落实第三期学前教育行动计划，积极发展普惠性幼儿园，推进义务教育向优质均衡发展，探索职业教育市场化运作模式，扶持规范社会力量办学，启动中高考改革准备工作。深化公立医院综合改革，推行“互联网+健康医疗”便民服务，推动民营医院建设。积极推进城乡客运一体化，更新新能源公交车辆63台，收回承包经营公交线路13条，公交车公营比例达到95%，稳步推进出租汽车行业改革。全面发展老龄、慈善、妇女儿童、退役军人、残疾人、地方志等事业，扎实做好外事侨务、宗教、气象、防震减灾等工作。

　　全面维护社会和谐稳定。深入推进平安辽源建设，完善立体化、智能型、全覆盖的社会治安防控体系。深入开展扫黑除恶专项斗争，深挖黑恶势力及其保护伞，彻查征地拆迁和拆违等工作中的乱象乱源。严格落实重大决策社会风险评估机制，畅通群众诉求表达、纠纷调处、权益保障机制，持续推进信访积案化解。切实加强安全生产监管，持续推进“冬春安全整治大会战”，强化“双重预防”机制建设，坚决遏制重特大事故发生。强化食品药品监管，坚决防控非洲猪瘟等重大动物疫病，确保群众生命财产安全。

　　（七）坚决克难攻坚，促进发展“向好”。

　　持之以恒打好打赢三大攻坚战，确保取得实效，实现更好发展。

　　积极防范化解重大风险。稳妥处理政府债务，强化属地风险处置责任，有序推进存量债务转换。充分发挥城投集团作用，通过完善土地收储、处置闲置资产、债务重组等方式降低债务存量，有效化解政府债务风险。高度重视重点企业债务和资金危机，积极争取银行等债权人支持，加快引进战略投资者，探索实施市场化债转股。争取省级纾困基金，加强政银企对接，避免因简单压贷、抽贷、断贷造成企业资金链断裂，坚决防止企业层面风险向经济层面、金融层面和社会层面传导。全面整治社会非法集资、企业信用债违约、P2P 网络借贷等风险点。

　　坚决打赢脱贫攻坚战。构建专项扶贫、行业扶贫、社会扶贫“三位一体”扶贫格局，突出产业扶贫引领，推动发展特色农业、纺织袜业、电子商务、乡村旅游等产业项目建设，补足补齐道路交通、村屯环境、公共服务等基础短板。突出行业扶贫统筹，完善“五点双享两救助”保障机制，加强教育、医疗等专项支持。突出社会力量支持，探索“百企进百村”助力精准脱贫等扶贫结对帮扶措施，扩大并发挥社会志愿者组织作用。严格对照“两不愁、三保障”要求，高质量实现3431人脱贫、10个贫困村退出目标，确保贫困人口全部脱贫。

　　加快解决环境突出问题。坚决抓好中央环保督察反馈问题整改，全面打好蓝天、碧水、青山、黑土“四个保卫战”。把东辽河流域治理作为标志性战役，确保到2020年实现基本消除劣五类水体目标。围绕水生态修复，加快3520.5公顷缓冲带、水源涵养林、河道涵养林建设，启动5.7万公顷退耕还林工程。围绕水环境治理，推动市、县、乡8个污水处理厂建成投入运营，如期完成仙人河截污干管迁移、仙人河清淤疏浚、雨污分流改造等控污减污工程。围绕水资源保护，加快引松入辽供水、工业节水、农业节水等引流节水项目建设，继续推进杨木水库等饮用水水源地治理项目。严格落实河长制和“一河一策”等管理措施，推动岸上岸下、上游下游系统性治理。加强大气污染防治，深入开展超标小锅炉、机动车尾气、城市扬尘、餐饮油烟、露天焚烧秸秆等专项整治，全年优良天数达到80%以上。全面开展采煤沉陷区、露天矿坑、矸石山等综合整治，逐步恢复生态功能。严格控制重点流域、重点区域环境风险项目，积极打造绿色产业群和生态集聚区。重拳打击违法案件，让破坏生态环境行为付出沉重代价。

　　（八）扩大开放合作，促进格局“向宽”。

　　坚持以“五个合作”为引领，积极融入全省规划布局，提高经济发展外向度。

　　强化产业互动合作。密切对接绍兴纺织袜业、新能源、绿色食品等重点产业，探索设计我市绍兴产业园，重点推动与大唐镇袜业园、嵊州市羲源南鹿梅花鹿产业、中盈志合镍氢电池等项目合作，加快显顺琵琶异地联合办学、现代职教园区与绍兴联合教学实训基地建设。利用与上海合作的产业转移促进中心平台，搭建产业互动、合作交流长效机制，加快承接医药健康等产业、技术和项目转移。

　　强化战略规划对接。主动融入长四辽吉松工业走廊和长辽梅通白敦医药健康产业走廊，沿线规划摆布新能源汽车配件、化药原料药等产业链项目，打造配套“双廊”核心产业基地。加强与“长春经济圈”城市互动，谋划推动科技、金融、有机农业、跨境电商等重点共赢合作项目。借助省级“1+N”战略合作框架和东博会平台，加强“一带一路”开放合作，构建国际友好城市联盟，建立常态化合作体系。

　　强化精准招商引资。全员动员招商引资，推行领导干部“包产业、包招商、包推进”制度，建立共享、会商和跟踪推进机制，完善招商引资签约项目考核管理、激励奖励办法，提高项目竣工率和达产率。突出长三角、珠三角、环渤海等重点区域，全面开展产业链招商、以商招商、驻点招商，探索中介招商、委托招商、高端智库招商，力争跟进对接、签约落地一批重大项目。

　　（九）打造服务政府，促进队伍“向上”。

　　锲而不舍、驰而不息地推进自身建设，努力打造人民满意政府。

　　坚持管理创新。结合政府机构改革，优化职能配置，理顺职责关系，积极构建系统完备、科学规范、运行高效的机构职能体系。加强工作流程监管，建立差异化绩效考核办法，全面实施项目化、工程化清单，形成有目标、有路径、有措施、有时限、有奖惩的工作推进机制。探索行业监管创新，严管重处违法违规行为，审慎包容对待新业态新模式。全面加强信用体系建设，扩大政务信用信息征集和应用范围，强化信用记录应用，让守信者处处受益，让失信者寸步难行。

　　坚持依法行政。深入贯彻法治政府建设实施纲要，认真履行宪法和法律赋予的职责，严格落实“权力清单”“责任清单”“负面清单”制度，确保行政权力始终在法治轨道上运行。自觉接受人大监督、政协监督和各方面监督，认真办理人大代表建议和政协提案。深化行政执法体制改革，完善执法监督和审计监督制度。严格执行政府工作规则，对重大决策严格实行前期广泛调研、专家综合论证、合法性审查、社会风险评估、集体讨论决定程序。深入推进“七五”普法，开展以宪法为核心的法治宣传教育，让崇尚法律、敬畏法律成为全社会共识。

　　坚持勤政为民。牢固树立“人民利益高于一切”的价值取向，把更多的财力、物力和精力集中到增进民生福祉上来，着力解决群众最关心、最直接、最现实的利益问题。大兴调查研究之风，深入到困难多、矛盾多的地方，听实话、访民情、找问题、解难事，始终把问政于民、问计于民贯穿工作始终，让政府的每项工作都体现广大人民群众的所需所盼，用真心真情践诺初心、温暖人心、赢得民心。

　　坚持转变作风。落实全面从严治党要求，牢固树立“四个意识”，坚定“四个自信”，做到“两个坚决维护”，严明政治纪律和政治规矩，营造风清气正、昂扬向上的政治生态。巩固“破五弊”“解放思想大讨论”成果，树立担当作为的政风导向，搭建施展才华的舞台平台，激发各级干部担当加快发展重任、担负改善民生重托、担起破解难题重担，真正打造一支政治过硬、能力过硬、作风过硬的公务员队伍。

　　坚持廉洁从政。严格落实党风廉政建设主体责任和“一岗双责”，认真落实中央八项规定及其实施细则精神，牢牢守住意识形态领域阵地。全面推进惩治和预防腐败体系建设，聚焦侵害群众切身利益的“微腐败”，盯紧征地拆迁、脱贫攻坚、惠民利民等重点领域，零容忍、出重拳，有腐必惩、有贪必肃，干净干事、严守底线，真正做到干部清正、政府清廉、政治清明。

　　各位代表，新时代赋予辽源新使命、新担当。让我们高举习近平新时代中国特色社会主义思想伟大旗帜，在市委的坚强领导下，进一步解放思想、坚定信心，务实重行、开拓创新，以新形象新担当新作为，奋力开创辽源创新转型、高质量发展新局面，以优异成绩迎接新中国成立70周年！
